# Evaluation of HIF-1α Expression in a Rat Glioma Model Using Intravoxel Incoherent Motion and R2* Mapping

**DOI:** 10.3389/fonc.2022.902612

**Published:** 2022-06-17

**Authors:** Dongdong Wang, Yiping Lu, Xuanxuan Li, Nan Mei, Pu-Yeh Wu, Daoying Geng, Hao Wu, Bo Yin

**Affiliations:** ^1^Department of Radiology, Huashan Hospital, Fudan University, Shanghai, China; ^2^Department of MR Research, GE Healthcare, Shanghai, China; ^3^Department of Dermatology, Huashan Hospital, Fudan University, Shanghai, China

**Keywords:** glioma, IVIM, hypoxia, HIF - 1α, R2* mapping

## Abstract

Accurate evaluation of HIF-1α levels can facilitate the detection of hypoxia niches in glioma and treatment decisions. To investigate the feasibility of intravoxel incoherent motion (IVIM) and R2* Mapping for detecting HIF-1α expression levels, sixteen rats with intracranial C6 gliomas were subjected to IVIM and R2* Mapping using a 7 Tesla MRI scanner. For each model, the brain tissue on the HIF-1α-stained slices was subdivided into multiple square regions of interest (ROIs) with areas of 1 mm^2^, for which HIF-1α expression was assessed by HALO software to form a maps of HIF scores with a 0–300 range. The IVIM and R2* Mapping images were processed to create maps of the D, D*, f and R2* that were then paired with the corresponding HIF score maps. The average D, D*, f, perfusion (f × D*) and R2* values were calculated for the ROIs in the tumor and normal brain regions with different HIF-1α levels and used in further analysis. In this study, the average tumor size of sixteen C6 model rats was 458 ± 46.52 mm^3^, and the 482 included ROIs consisted of 280 tumoral and 202 normal ROIs. The average HIF score for the tumor regions was significantly higher than normal brain tissue (p < 0.001), and higher HIF scores were obtained for the central part of tumors than peripheral parts (p=0.03). Compared with normal brain tissues, elevated perfusion and f values were observed in tumor regions (p = 0.021, 0.004). In tumoral ROIs, the R2* values were higher in the group with high HIF-1α expression than in the group with low HIF-1α expression (p = 0.003). A correlation analysis revealed a positive correlation between the R2* value and HIF scores (r = 0.43, p < 0.001) and a negative correlation between D* and the HIF scores (r = -0.30, p = 0.001). Discrepancies in HIF-1α expression were found among different intratumoral areas, and IVIM and R2* Mapping were found to be promising means of noninvasive detection of the distribution and expression level of HIF-1α.

## Introduction

Glioma is the most common and malignant primary brain tumor in humans and has a specific histological hallmark of microvascular proliferation (1). Dysfunctional neoangiogenesis is believed to contribute to a hypoxic microenvironment in glioma and resistance to chemoradiotherapy (2–5). Molecular responses to hypoxia are predominantly mediated by increased activity of hypoxia-inducible factors (HIFs), predominantly HIF-1α (5). Accurate evaluation of HIF-1α levels can facilitate the detection of hypoxia niches in glioma and treatment decisions. Traditionally, invasive methods, including oxygen electrodes and biopsy-derived techniques, have been used to assess HIF-1α expression in glioma, but have accuracies that largely depend on tissue sampling and cannot reflect the oxygenation status of the whole tumor due to the heterogeneous distribution of tumor hypoxia (6, 7). Noninvasive and convenient methods for assessing HIF-1α expression and accurate assessment of whole-tumor hypoxia would be extremely useful.

Two noninvasive and contrast-free functional MRI techniques, named intravoxel incoherent motion (IVIM) and R2* Mapping, have made it possible to visualize hypoxic regions. IVIM was first introduced by Le Bihan et al. (8) and can be used to distinguish the incoherent motion of water molecules within capillaries from extravascular molecular diffusion. The true diffusion coefficient (D), pseudodiffusion coefficient (D*), and perfusion fraction (f) were calculated by applying a biexponential model to multiple b-value diffusion images. Previous reports have demonstrated that IVIM could serve as an important guide for the quantitative assessment of HIF-1α expression in solid prostate cancer (9). The other technique, R2* Mapping, has been used to determine tissue oxygen bioavailability by measuring alterations in deoxyhemoglobin, an endogenous MR imaging marker with the property of paramagnetism (10). It was proven that R2* Mapping can detect alterations in oxygenation status, and a significant correlation between R2* and HIF-1α expression in the kidney was also reported (11). To date, no research has been performed on evaluating the usage of IVIM and R2* Mapping to visualize HIF-1α expression in glioma.

Therefore, the aim of this study was to use IVIM and R2* Mapping parameters (D, D*, f, perfusion and R2*) to determine HIF-1α expression levels in rat glioma models, investigate the feasibility of these two potential hypoxia imaging methods, and evaluate the corresponding application potential for further clinical practice.

## Materials and Methods

All studies were approved by the Institutional Animal Care and Use Committee of the Medical School of Fudan University.

### Rat Model

Male Wistar rats (age 6–10 weeks, n = 16) were anesthetized by intraperitoneal injection of medetomidine (0.4 mg/kg) and ketamine (70 mg/kg). Approximately 1 × 10^6^ cells in 5 μl were stereotactically injected into the basal ganglia (3 mm left of the bregma at a 4.5-mm depth in the brain). Metamizol (50 mg/kg) was injected subcutaneously to prevent postoperative pain. The rats were monitored for mood behavior and activity levels throughout the entire experiment using the volume of droppings as a surrogate measurement.

### MRI Examination

Seven days after the rats were injected with tumor cells, T2WI MRI scanning was performed to evaluate the volume of each tumor every two days. On the 14th day after being injected with tumor cells, the rats were anaesthetized by intraperitoneal injection of medetomidine and ketamine, and subsequently intubated and maintained under anesthesia with isoflurane using active ventilation. To improve the stability and repeatability of image acquisition, the rat body temperature was maintained at 37.0°C using a heating pad, and the respiratory rate was maintained at 50–70 breaths per minute using a mixture of air containing 1%–2% isoflurane to minimize breathing artifacts while sustaining normal oxygen delivery.

MR imaging was performed on a 7.0 T small-animal MR unit (Bruker PharmaScan, Ettlingen, Germany) equipped with a transmit-receive quadrature volume coil with an inner diameter of 3.8 cm. Anatomical T2-weighted images were obtained using a 2D fast spin echo sequence (three orthogonal directions; TR = 3000; TE = 33 ms; 7 echoes; field of view (FOV) = 25.6×25.6mm^2^; matrix size = 96×96; slice thickness = 0.5 mm; 20 slices). IVIM images were acquired using a free-breathing single-shot echo-planar imaging pulse sequence with 10 b values (TR = 2000 ms; TE = 23 ms; FOV = 25.6×25.6 mm^2^; matrix size = 96×96; slice thickness = 1 mm; 10 slices; b = 0, 25, 50, 75, 100, 150, 200, 300, 1000, 1500, 2000 mm^2^/s; scan time = 3 min 20 seconds). R2* Mapping was performed with a 3D spoiled gradient-echo sequence (TR = 160 ms; TE = 3.4, 9.3, 15.2, 21.2, 27.1, 33, 38.9, 44.8, 50.7, 56.6, 62.5, 68.5, 74.4, 80.3, 86.2, 92.1 ms; FOV =25.6×25.6 mm^2^; matrix size=96×96; slice thickness = 1 mm; 10 slices; NEX = 2; scan time = 6 min 20 seconds). The IVIM and R2* Mapping sequences were registered with the T2WI images.

### Postprocessing

The IVIM-DWI data was postprocessed using Functool-MADC software on an Advantage Workstation (Version 4.6; GE Health care, Milwaukee, WI, USA). An IVIM-DWI biexponential model was defined as SI/SI_0_ = (1−f) × exp(−bD) + f × exp(−bD*), where SI_0_ is the mean signal intensity of the ROI under consideration at b = 0 mm^2^/s, and SI is the signal intensity at higher b values. D denotes the diffusion coefficient of water molecules (the true diffusion coefficient), D* represents the microcirculation perfusion (pseudodiffusion) coefficient, f is the perfusion fraction, and f multiplied by D* represents the perfusion (12). We also obtained R2* values from the R2* Mapping images using the Functool-R2* mapping software on the Advantage Workstation(Version 4.6; GE Health care, Milwaukee, WI, USA) as well. The R2* maps for each tumor were calculated and R2* values were calculated as the reciprocal of the T2* values.

### Immunohistochemistry

The rats were sacrificed after the MRI scans were taken. The rats were overdosed with ketamine and medetomidine and then transcardially perfused with 4% paraformaldehyde in PBS. The rat brains were removed, postfixed in 4% paraformaldehyde in PBS overnight, dissected and embedded in paraffin. Five-micrometer brain sections were cut using a sliding microtome and processed.

The sections were stained with hematoxylin and eosin as per standard protocol. The stained slides were deparaffined in xylene, dehydrated through graded alcohol concentrations, and washed in Tris buffer (pH = 7.0). Antigen retrieval was performed by heat mediation in citrate buffer (pH = 6.2) for 1 hour at 90°C and then blocked using 5% skim milk. The sections were incubated with a rabbit monoclonal anti-human HIF-1α antibody (1/50, ab51608, Abcam, Cambridge, UK) for 16 hours at 4°C. Undiluted horseradish peroxidase-conjugated antirabbit IgG (ab150081, Abcam) was used as the secondary antibody. All specimens were investigated by HALO image analysis software.

### MR and Pathological Analysis

The largest tumor section on coronal T2WI was selected, and the corresponding coronal pathological slices were measured with electronic calipers at the largest cross-sectional area of the lesion. Subsequently, the corresponding slices of the IVIM and R2* Mapping images were selected.

The HIF‐1α scores, including the percent of positive HIF-1α cells and coloring strength, for all the rectangular ROIs were automatically evaluated by HALO image analysis software using the IHC v1.6 algorithm module (Indica Labs, NM) (13). All the ROIs had an area of 1 mm^2^ (14). The scores for the coloring strength and percentage of positive cells were multiplied together to produce final HIF-1α scores with a 0-300 range. An intratumoral region with a score over 150 was considered a region with high HIF-1α expression, whereas a score less than 150 was considered to indicate a region with low HIF-1α expression. The inclusion criteria for ROIs was as follows: (a) the ROI contained only tumor or normal tissue and (b) the proportion of the components of vessels, necrosis or calcification was less than 25%, without air. An ROI with a center less than 1 mm from the edge of the tumor was classified as a peripheral intratumoral part and was otherwise classified as a central intratumoral part.

**Figure 1:**
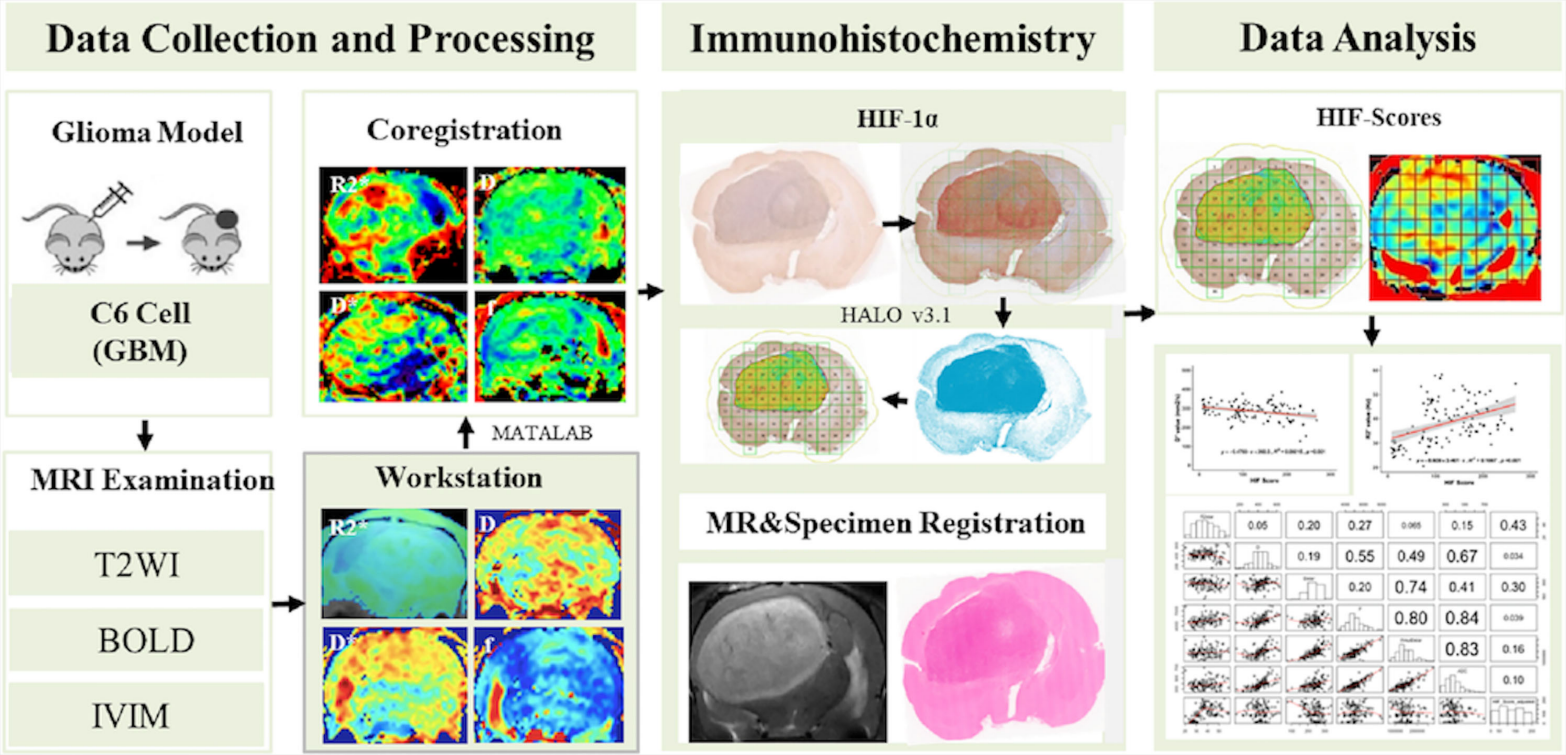
Workflow of the study.

An in-house MATLAB (Mathworks, Natick, MA, USA) script was used to perform a quantitative analysis. The IVIM/R2* Mapping images were subdivided into multiple ROIs corresponding to the subdivision of the corresponding pathological slices. The imaging parameters of all the areas were automatically output. The imaging parameters and corresponding HIF-1α scores were recorded for statistical analyses. The basic workflow of our study is illustrated in **Figure 1**.

### Statistical Analysis

Statistical analysis was performed using R software (Version 3.5.3; R Foundation for Statistical Computing, Vienna, Austria). The imaging parameters for the low- and high-expression groups were compared using the Mann–Whitney U-test. A p value less than 0.05 was considered statistically significant. The Spearman correlation coefficient was used to assess the relationships between the imaging parameters, including the D, D*, f, perfusion and R2* values and the HIF-1α scores for the tumor and normal tissue on the contralateral side. A simple linear regression analysis was performed to illustrate the relationship between imaging parameters and HIF-1α expression.

## Results

In this study, the average tumor size of sixteen rats was 458 ± 46.52 mm^3^, and the 482 included ROIs consisted of 280 ROIs of tumor tissue and 202 ROIs of normal brain tissue.

### 1. Tumor Tissue vs. Normal Brain Tissues

The HIF-1α expression level in tumor tissue was higher than that in normal tissue (123.73 ± 70.18 vs. 21.13 ± 19.14, p < 0.001), and the f and perfusion values of the tumor tissue were also higher than those of normal tissue (46.29 ± 2.75 vs. 51.09 ± 8.58, p = 0.004; 1277.09 ± 228.64 vs. 1463.88 ± 378.67, p = 0.021). There was no significant difference between the R2*, D, and D* values of tumor and normal tissues (**Table 1**).

**Table 1:** Mean parameter values for tumor and normal brain tissues.

Imaging/Pathological Parameter	Normal Brain Area (Mean ± SD, N = 202)	Tumor Region (Mean ± SD, N = 280)	P
R2* (Hz)	36.74 ± 9.35	38.24 ± 8.76	0.502
D (×10^-6^ mm^2^/s)	414.21 ± 53.38	419.50 ± 81.97	0.721
D* (×10^-6^ mm^2^/s)	2755.02 ± 441.42	2850.23 ± 444.48	0.373
f (%)	46.29 ± 2.75	51.09 ± 8.58	0.004*
Perfusion (×10^-6^ mm^2^/s)	1277.09 ± 228.64	1463.88 ± 378.67	0.021*
HIF-Score	21.13 ± 19.14	123.73 ± 70.18	<0.001*

* and bold values means p<0.05 was considered statistically significant.

### 2. The Spatial Distribution of Pathological and Imaging Features

**Figure 2:**
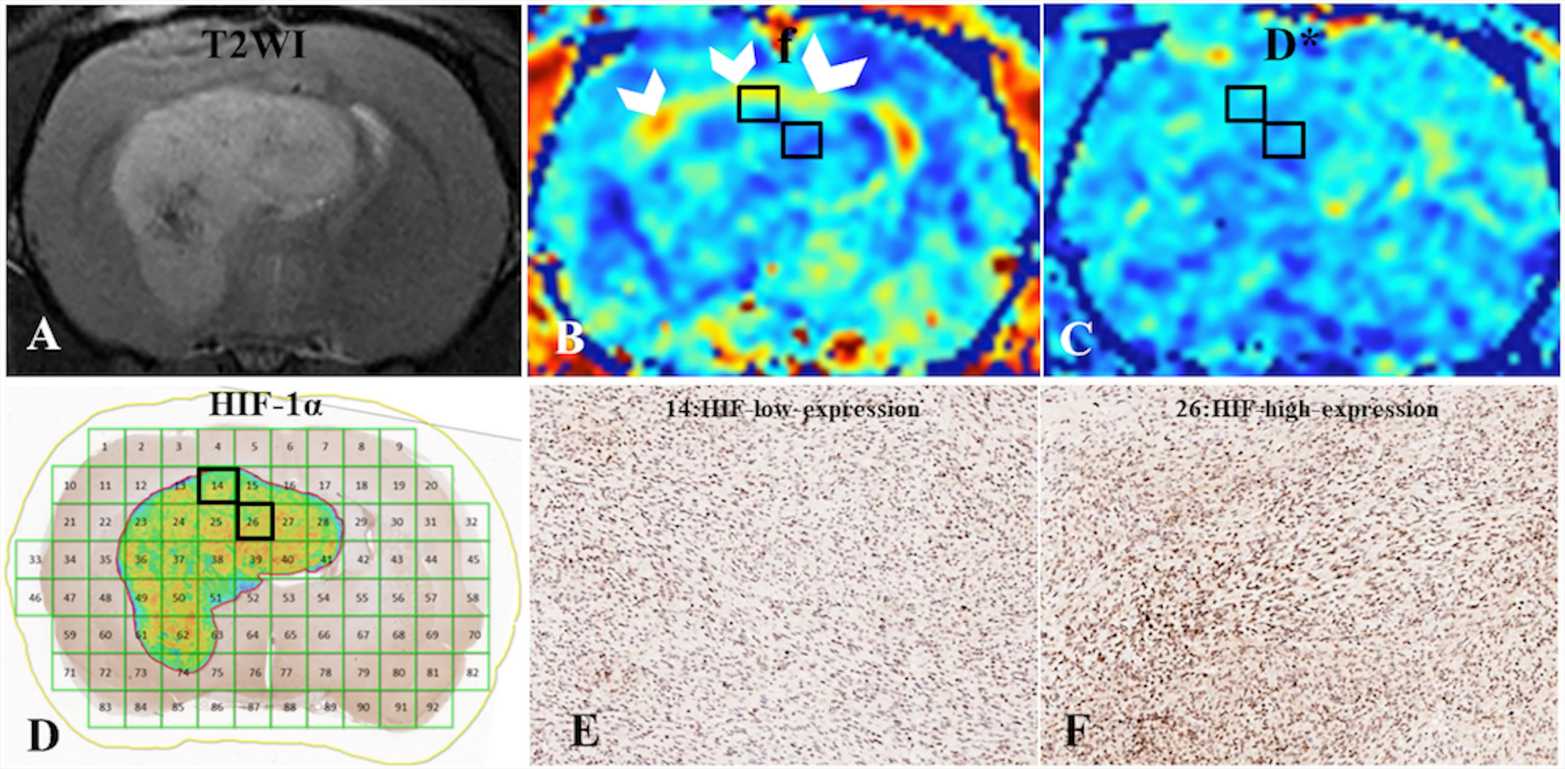
**(A)** C6-cell glioma tissue was observed in a T2WI image. **(B, C)** f and D* maps. **(D)** ROI locations in the tumor area correspond to 14 and 26 (HIF-1α map), for which the f values were 59% and 47%, respectively; the D* values were 341.69 ×10-6 mm2/s and 281.41 ×10-6 mm2/s, respectively; and the HIF-scores were 122.89 and 174.12, respectively. A high f value was found for the peripheral intratumoral tissue (white arrow). **(E, F)** Immunohistochemical staining images (× 400) showed low and high expression of HIF-1α in the tumor for ROIs 14 and 26, respectively, on the HIF-1α map.

HIF-1α expression in the central intratumoral area was higher than that in the peripheral areas (137.17 ± 76.23 vs. 108.77 ± 59.98, p = 0.03), and the perfusion, f, and D* values in the central intratumoral area were lower than those in the peripheral areas (1385.03 ± 427.17 vs. 1551.65 ± 296.05, p = 0.003; 50.00 ± 9.87 vs. 52.31 ± 6.76, p = 0.026; 2749.23 ± 487.95 vs. 2962.53 ± 363.06, p = 0.044) (**Figure 2**). There was no significant difference in the spatial distributions of the R2* or D values between the tumor and normal tissue (**Table 2**).

**Table 2:** Mean parameter values for the spatial distribution of pathological and imaging features.

Imaging/Pathological Parameter	Central Part of Tumor (Mean ± SD, N = 171)	Peripheral Part of Tumor (Mean ± SD, N = 109)	P
R2* (Hz)	38.23 ± 8.64	38.25 ± 8.98	0.989
D (×10^-6^ mm^2^/s)	415.66 ± 78.99	423.77 ± 85.72	0.605
D* (×10^-6^ mm^2^/s)	2749.23 ± 487.95	2962.53 ± 363.06	0.044*
f (%)	50.00 ± 9.87	52.31 ± 6.76	0.026*
Perfusion (×10^-6^ mm^2^/s)	1385.03 ± 427.17	1551.65 ± 296.05	0.003*
HIF-Score	137.17 ± 76.23	108.77 ± 59.98	0.030*

* and bold values means p<0.05 was considered statistically significant.

### 3. The Distribution of Imaging Parameters in Groups With High and Low HIF-1α Expression

**Figure 3:**
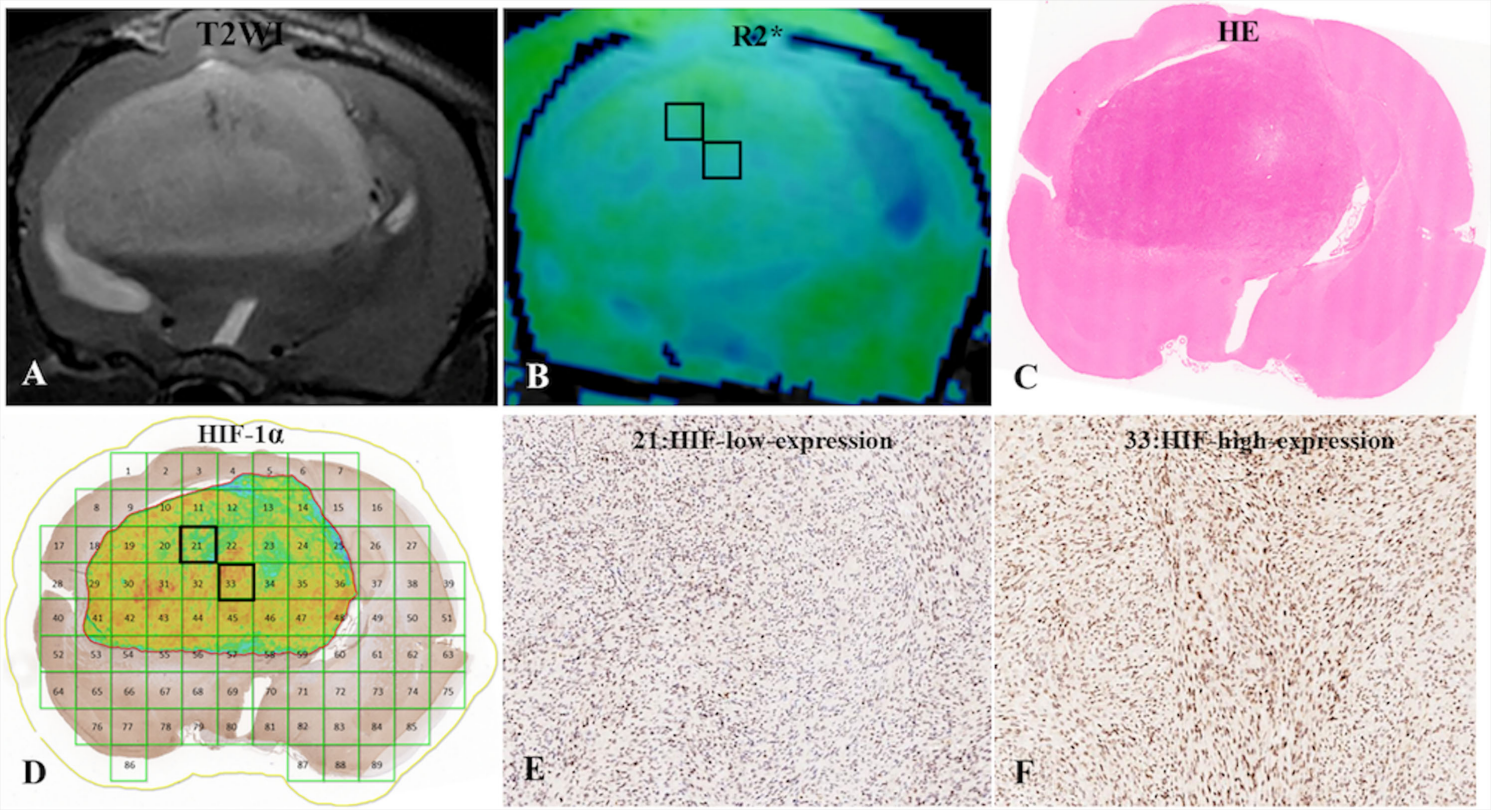
**(A)** C6-cell glioma tissue was observed in a T2WI image. **(B)** the post-processing R2* value of c6-cell glioma. **(C)** H&E staining. **(D)** ROI locations in the tumor area on the R2* map correspond to 21 and 33 (HIF-1a map), for which the R2* values were 33.50 Hz and 45.87 Hz, respectively, and the HIF-scores were 104.87 and 200.95, respectively. **(E, F)** Immunohistochemical staining images (× 400) showed low and high expression of HIF-1a in the tumor for ROIs 21 and 33, respectively, on the HIF-1a map.

The mean R2* was higher in the group with high HIF-1α expression than in the low-expression group (40.95 ± 6.46 vs. 36.49 ± 9.62, p = 0.003) (**Figure 3**). There was no significant difference in the D, f, and D* values for the intratumoral areas with high- and low-expression of HIF-1α (**Table 3**).

**Table 3:** Mean parameter values for the distribution of imaging parameters in high- and low-expression groups.

Imaging Parameter	Regions with High HIF-1a Expression (Mean ± SD, N = 143)	Regions with Low HIF-1a Expression (Mean ± SD, N = 137)	P
R2* (Hz)	40.95 ± 6.46	36.49 ± 9.62	0.003*
D (×10^-6^ mm^2^/s)	415.87 ± 77.37	421.85 ± 85.29	0.702
D* (×10^-6^ mm^2^/s)	2746.46 ± 533.77	2917.34 ± 364.43	0.106
f (%)	50.91 ± 8.04	51.21 ± 8.97	0.856
Perfusion (×10^-6^ mm^2^/s)	1408.84 ± 411.43	1499.49 ± 354.45	0.233

* and bold values means p<0.05 was considered statistically significant.

### 4. Regression Analysis Between the HIF Score and Imaging Parameters

**Figure 4:**
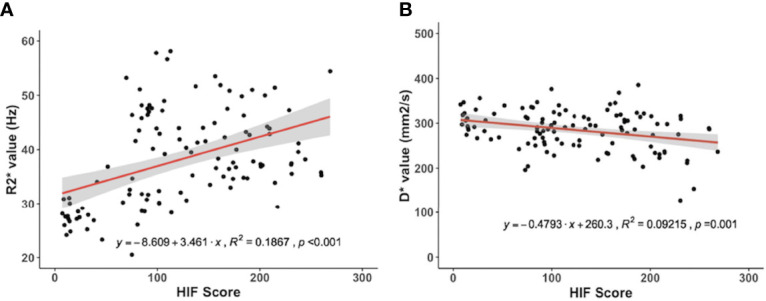
Correlations for the hypoxia‐inducible factor 1α (HIF‐1α) with the pseudodiffusion coefficient (D*) and the apparent transverse relaxation rate (R2*). **(A)** HIF‐1α correlated positively with the R2* value (r = 0.43, p < 0.001). **(B)** HIF‐1α correlated negatively with D* (r = -0.30, p = 0.001).

A Pearson correlation analysis revealed a negative correlation between D* and the HIF-1α expression levels (r = -0.30, p = 0.001). There was a positive correlation between the R2* value and the HIF-1α expression level (r = 0.43, p < 0.001) (**Figure 4**). There was no significant correlation between the HIF-1α expression level and the D, f and perfusion values.

## Discussion

Hypoxia is an important microenvironmental feature of glioma that can be categorized into chronic and acute hypoxia. Chronic or “diffusion-limited” hypoxia applies to tumor cells at a distance from blood vessels and can be detected by pimonidazole staining (15). “Acute” or “transient” hypoxia results from local fluctuations in blood perfusion and the oxygen supply and can be detected by HIF-1α staining: HIF-1α was found to be more diffusely distributed in tumors than pimonidazole. After 5 hours of hypoxia exposure, HIF-1α reaches maximal expression levels and returns to basal levels at 12 hours (16). Hypoxia is a clinical problem that contributes to therapy resistance, and acute hypoxia may be particularly dangerous because high HIF-1α expression upregulates canonical stem cell genes and facilitates plasticity toward a stem-like state. Especially in glioblastoma (GBM), the hypoxic microenvironment may enable GBM cells under certain inhibitor treatments to maintain a stem-like phenotype (17). Thus, the accurate detection of HIF‐1α expression in glioma facilitates making individual treatment plans, especially regarding the usage of anti-hypoxia drugs. In this study, we found satisfactory feasibility for using the noninvasive methods of R2* Mapping and IVIM imaging to detect HIF-1α expression in glioma.

We quantitatively analyzed immunohistochemical images using HALO software to calculate a density heatmap of HIF-1α, including quantitatively determining the staining intensity and the corresponding percentage of positive cells. The automatic calculation of HIF scores prevents the introduction of bias from different pathologists calculating HIF scores. In addition, the corresponding parameter values of all the areas of the MRI images were automatically output by an in-house MATLAB script, which also circumvents the introduction of bias from manually drawing ROIs. Christen et al. (18, 19) drew an ROI around a whole tumor and compared the imaging parameters of the tumor with those of the contralateral striatum, neglecting GBM heterogeneity. In this study, we divided the tumor into multiple rectangular ROIs of equal sizes to prevent the introduction of bias from tumor heterogeneity and increase the accuracy of the results.

R2* Mapping is a noninvasive method that offers a unique advantage in being able to realize dynamic and repeated detection of altered tissue oxygenation without the use of ionizing radiation and has exhibited good reproducibility in the kidneys of rabbit models (10, 11, 20, 21). In this study, no significant difference was found between the R2* values of tumor and uninvolved contralateral brain tissue, which was consistent with the results of a previous study (18). However, as mentioned above, we compared the ROIs in intratumor tissue with high and low expression of HIF-1α and found the R2* value was useful for determining the HIF-1a distribution. This result can be explained by the dependence of R2* on the deoxyhemoglobin content, where increased paramagnetic deoxyhemoglobin generation in tumors contributed to increased R2* values. Due to tumor vasculature destruction or increased oxygen demand for tumor regeneration, the R2* values of intratumoral tissue zones with high HIF expression were higher than for those with low HIF expression. In addition, a significant positive correlation was observed between R2* and HIF-1α in the glioma model, suggesting that R2* Mapping can be used to detect different levels of glioma tissue oxygenation.

In fact, hypoxia is promoted by high oxygen consumption in tumor regions with increased cellularity and decreased oxygen supply because of impaired vascular structure. Our results showed higher HIF-1α expression in the central tumor area than in peripheral tumor parts and normal tissue, and the perfusion, f, and D* values in the central tumor parts were lower than those in the peripheral parts. However, no significant difference between the D values of the central and peripheral tissue was found in this study. The D value is a measure of the diffusion of water molecules and the cell density. Thus, the higher HIF-1α expression in central intratumor tissue may be caused by impaired vessels, which is consistent with the perivascular and vascular-invasive niches of glioblastoma (22).

The results of this study also showed that the IVIM parameters could be used to detect HIF-1α expression levels in glioma tissue. The negative correlation between D* and HIF-1α expression levels could be attributed to D* mainly being a measure of microcirculation perfusion. A high D* corresponds to high supply of blood and oxygen. A disorganized and functionally impaired vasculature in glioma can contribute to decreasing D*, and the metabolic demand of tumor cells leads to increased HIF-1α expression. However, in this study, D and f were poor indicators for assessing HIF-1α expression because technical limitations, such as data instability and a low SNR, made it difficult to measure f and D accurately, which may be the main reason for why these parameters were not useful for predicting HIF-1α expression (23).

Our study had several limitations. First, IVIM and R2* Mapping could not be used to measure tissue oxygen levels directly. The R2* and D values were not in agreement with the PtO_2_ levels and were compromised by the absence of direct quantification (18, 21, 24). Second, we did not observe dynamic changes in R2* and the IVIM parameters during glioma development, and further study is necessary before this technique can be extensively used in clinical practice.

In conclusion, there was heterogeneity of HIF-1α expression in glioma tissue, and IVIM and R2* Mapping were found to be promising methods for the noninvasive detection of the distribution and expression level of HIF-1α. Both the D* parameter of IVIM and the R2* parameter of R2* Mapping were significantly correlated with HIF-1α expression and could be useful for the assessment and mapping of HIF-1a expression.

## Data Availability Statement

The raw data supporting the conclusions of this article will be made available by the authors, without undue reservation.

## Ethics Statement

The animal study was reviewed and approved by Institutional Animal Care and Use Committee of the Medical School of Fudan University.

## Author Contributions

Author PW was employed by company GE Healthcare.

The remaining authors declare that the research was conducted in the absence of any commercial or financial relationships that could be construed as a potential conflict of interest.

## Funding

This study has received funding by Youth Program of National Natural Science Foundation of China (Fund No. 81901697), the Youth Medical Talents –Medical Imaging Practitioner Program [Grant number SHWRS(2020)_087] to YL, Shanghai Sailing Program (Grant No. 21YF1404800) to XL, Youth Program of Special Project for Clinical Research of Shanghai Municipal Health Commission Health industry(Grant No. 20204Y0421) to DW and Science  and Technology Innovation Action Plan of Shanghai Science and Technology Commission(Grant No. 22S31905900) to BY.

## Conflict of Interest

The authors declare that the research was conducted in the absence of any commercial or financial relationships that could be construed as a potential conflict of interest.

## Publisher’s Note

All claims expressed in this article are solely those of the authors and do not necessarily represent those of their affiliated organizations, or those of the publisher, the editors and the reviewers. Any product that may be evaluated in this article, or claim that may be made by its manufacturer, is not guaranteed or endorsed by the publisher.
